# Serum 25-Hydroxyvitamin D Concentrations and Season-Specific Correlates in Japanese Adults

**DOI:** 10.2188/jea.JE20100161

**Published:** 2011-09-05

**Authors:** Akiko Nanri, Leng Huat Foo, Kazutoshi Nakamura, Ai Hori, Kalpana Poudel-Tandukar, Yumi Matsushita, Tetsuya Mizoue

**Affiliations:** 1 Department of Epidemiology and Prevention, International Clinical Research Center, National Center for Global Health and Medicine, Tokyo, Japan; 2 School of Health Sciences, Universiti Sains Malaysia, Kelantan, Malaysia; 3 Department of Community Preventive Medicine, Niigata University Graduate School of Medical and Dental Sciences, Niigata, Japan

**Keywords:** 25-hydroxyvitamin D, Japanese, lifestyle factors, vitamin D intakes

## Abstract

**Background:**

Several lines of evidence indicate an important role for vitamin D in the prevention of a range of diseases. Blood vitamin D levels show clear seasonal variation; however, data on the determinants of vitamin D status for each season are limited. We investigated the association between lifestyle and serum vitamin D concentration by season in Japanese workers.

**Methods:**

Subjects were 312 men and 217 women aged 21 to 67 years who worked in municipal offices in Northern Kyushu, Japan and participated in a periodic checkup in July or November. Multiple linear regression analysis was used to examine the association between serum 25-hydroxivitamin D concentrations and lifestyle factors for each season.

**Results:**

Mean serum 25-hydroxyvitamin D concentration was 27.4 ng/ml (68.4 nmol/L) and 21.4 ng/ml (53.4 nmol/L) for workers surveyed in July and November, respectively (*P* < 0.001); the prevalence of vitamin D deficiency (<20 ng/ml) was 9.3% and 46.7%, respectively (*P* < 0.001). In November, dietary vitamin D intake (in both sexes) and nonsmoking and physical activity (in men) were significantly associated with higher concentrations of serum 25-hydroxyvitamin D. In summer, fish/shellfish intake was associated with higher serum 25-hydroxyvitamin D concentrations in women.

**Conclusions:**

Vitamin D deficiency is common in Japanese workers during seasons with limited sunlight. The lifestyle correlates of favorable vitamin D status in November were physical activity, dietary vitamin D intake, and nonsmoking.

## INTRODUCTION

Vitamin D plays an important role in maintaining musculoskeletal health, but accumulating evidence indicates that it has a protective role against a range of diseases, including cancer, cardiovascular diseases, autoimmune diseases, infectious diseases, and diabetes.^1^ Vitamin D status (best determined by measuring blood 25-hydroxyvitamin D concentration) is largely due to exposure to sunlight, which induces vitamin D production in the skin.^2^ Accordingly, vitamin D levels show clear seasonal variation: they are highest in late summer and lowest in late winter or early spring.^2^

The identification of determinants of vitamin D levels is a prerequisite for an effective intervention. Studies have shown that vitamin D concentrations are lower in women than in men,^3^^–^^9^ decrease with aging^4^^,^^8^^–^^12^ and high body mass index (BMI),^4^^,^^6^^,^^8^^,^^13^^–^^18^ and increase with physical activity,^6^^,^^8^^,^^12^^,^^14^^,^^18^ outdoor activity,^3^^,^^8^^,^^9^ and intake of vitamin D,^4^^,^^6^^,^^14^^,^^15^^,^^18^^–^^20^ vitamin D supplements,^3^^,^^4^^,^^8^^,^^9^^,^^13^^–^^15^^,^^17^^,^^18^^,^^21^ and fish.^3^^,^^6^^,^^9^^,^^21^^–^^23^ Several of these studies adjusted for season of blood collection as a confounder.^9^^,^^12^^,^^14^^,^^16^^,^^18^^,^^21^ However, given the large contribution of sun-induced vitamin D production in the skin, the effect of lifestyle factors on vitamin D status might be better assessed according to season. To our knowledge, 6 studies have investigated the association between lifestyle factors and blood vitamin D levels by season,^3^^,^^4^^,^^6^^,^^8^^,^^19^^,^^24^ and only 1 examined the season-specific association among Asian populations.^24^ Moreover, no study of this association was conducted in an occupational health setting. Here, we examined the seasonality of the association of serum 25-hydroxyvitamin D concentrations with demographic and lifestyle factors in a Japanese working population.

## METHODS

### Subjects

Details of the study design have been described elsewhere.^25^^,^^26^ In short, the participants were recruited from among the employees of 2 municipal offices in Northeast Kyushu, Japan (33.4–33.5° N). Of 601 eligible workers, 547 (91%) aged 21 to 67 years agreed to participate in a health survey at the time of the employee health examination, which was conducted in July (workplace A) or November (workplace B) 2006. Participants were asked to donate a sample of venous blood, complete 2 questionnaires (one for lifestyle and health condition and another for diet), and provide us with their health checkup data. After further exclusion of 18 subjects with missing information for any of the variables investigated in the present analysis, 529 participants (workplace A, 161; workplace B, 368) remained. The protocol of the study was approved by the ethics committee of the National Center for Global Health and Medicine, and written informed consent was obtained from each participant before the study.

### Serum 25-hydroxyvitamin D

Serum 25-hydroxyvirtamin D concentrations were measured at an external laboratory by using a competitive protein binding assay (Mitsubishi Chemical Medience Corporation, Tokyo, Japan). The intra-assay coefficients of variation were 7.3% to 10.3%. Vitamin D deficiency was defined as a serum 25-hydroxyvitamin D concentration lower than 20 ng/ml (50 nmol/L).^1^ This cutoff point is accepted by most experts and has been adopted in numerous studies.^1^^,^^27^

### Lifestyle and body measurements

Body height was measured to the nearest 0.1 cm with the subject standing without shoes. Body weight in light clothes was measured to the nearest 0.1 kg. BMI was calculated as body weight in kilograms divided by the square of body height in meters. In the survey questionnaire, non-job physical activity was defined as minutes per day spent walking or cycling during commuting to and from work and weekly hours engaged in each of 5 different leisure activities (walking; low-, moderate-, and high-intensity activities; and gardening). The sum of time spent on all non-job activities was expressed in hours per week.

Dietary habits during the preceding 1 month were assessed using a validated, brief, self-administered diet history questionnaire (BDHQ),^28^ which contains 56 foods and beverages commonly consumed in Japanese populations. Dietary intakes of energy and selected nutrients, including vitamin D, were estimated using an ad hoc computer algorithm for the BDHQ, with reference to the Standard Tables of Food Composition in Japan.^29^^,^^30^ Because few subjects took dietary supplements on a weekly basis (multivitamins, 3.7%; vitamin D, 0.2%; and multiminerals, 1.1%), dietary supplementation was not incorporated into the analysis. Because the major sources of dietary vitamin D intake in Japanese populations are fish/shellfish (79%) and egg (9%),^31^ we also examined the association with these intakes in addition to vitamin D intake. In a validation study of the BDHQ against 16-day weighed dietary records among 92 men and 92 women aged 31 to 76 years, the Pearson correlation coefficients for vitamin D intake were 0.38 in men and 0.40 in women, and the Spearman correlation coefficient for fish/shellfish intake and egg intake were 0.29 and 0.55, respectively, in men and 0.41 and 0.32 in women.^28^^,^^32^

### Statistical analysis

The independent *t*-test (for continuous variables) and chi-square test (for categorical variables) were used to assess the statistical significance of differences between sexes and the workplaces where the survey was conducted in different seasons. Factors considered in the analysis were sex, age (<35, 35–44, or ≥45 years), BMI (<21, 21–22.9, 23–24.9, or ≥25 kg/m^2^), occupation (office work or others), smoking status (nonsmoker or current smoker), alcohol consumption (nondrinker or drinker consuming either <20 or ≥20 g ethanol/day), non-job physical activity (none, <2, or ≥2 hours/week), dietary vitamin D intake (tertile), fish/shellfish intake (tertile), and egg intake (tertile). Vitamin D intake was adjusted for total energy intake using the density method (intake per 1000 kcal). We confirmed that when a factor was entered into the multivariate model as a continuous variable, the association between other factors and serum 25-hydroxyvitamin D did not substantially change. To examine the season-specific association between these lifestyle factors and serum 25-hydroxyvitamin D concentrations, we used multiple linear regression analysis to calculate the means and standard errors of serum 25-hydroxyvitamin D concentrations for each exposure category, with adjustment for other covariates in each workplace. We examined effect modification by adding to the model an interaction term of an explanatory variable and others in each survey season. Because we detected significant interactions between sex and some factors in each season, we conducted stratified analyses by sex in each workplace. A 2-sided *P* value less than 0.05 was considered to indicate statistical significance. All analyses were done with Statistical Analysis System (SAS) software version 9.1 (SAS Institute, Cary, NC, USA).

## RESULTS

The characteristics of the study participants are shown in Table 1. In both seasons, serum 25-hydroxyvitamin D concentrations were significantly higher in men than in women; the mean values of serum 25-hydroxyvitamin D for men and women were 28.0 and 26.3 ng/ml, respectively, in summer and 22.9 and 19.4 ng/ml in late autumn. In addition, serum 25-hydroxyvitamin D concentrations among both men and women which were much lower in workplace B (surveyed in November) than in workplace A (surveyed in July). Moreover, men in workplace A had a higher BMI and consumed more eggs than did those in workplace B. Factors other than vitamin D concentration and egg intake did not significantly differ between workplaces. The distribution of serum 25-hydroxyvitamin D concentration by workplace, season, and sex is shown in the Figure. By sex, 35% of men and 62% of women surveyed in late autumn had vitamin D deficiency (<20 ng/ml), whereas only approximately 10% of participants surveyed in summer had vitamin D deficiency.

**Figure.**
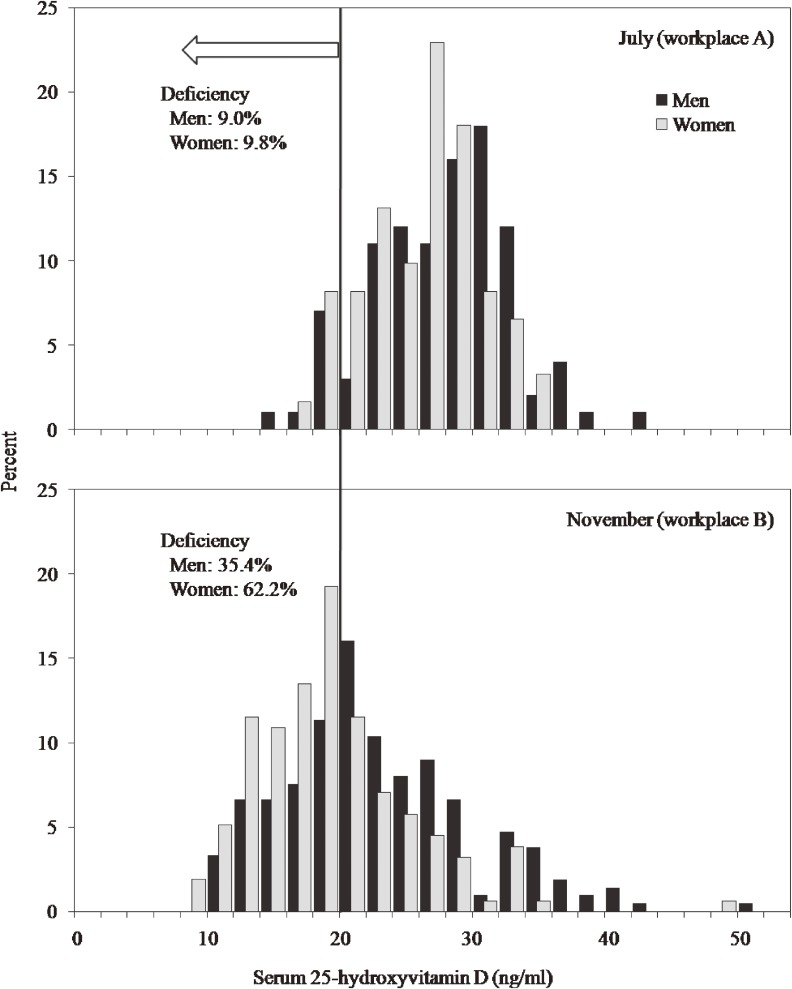
Distribution of serum 25-hydroxyvitamin D concentrations by survey season and sex

**Table 1.** Characteristics of study subjects

	Workplace A (July)			Workplace B (November)			*P* value^b^	
			
	Men	Women	*P* value^a^	Men	Women	*P* value^a^	Men	Women
No. of participants	100	61		212	156			
Age (years)	44.8 ± 10.8	40.5 ± 9.8	0.01	44.3 ± 10.8	41.2 ± 10.8	<0.01	0.67	0.68
Body mass index (kg/m^2^)	24.3 ± 3.5	20.7 ± 2.8	<0.01	23.2 ± 3.1	21.2 ± 3.3	<0.01	<0.01	0.27
Occupation (office work, %)	93.0	55.7	<0.01	87.3	71.2	<0.01	0.13	0.03
Cigarette smoking (current, %)	43.0	3.3	<0.01	44.3	2.6	<0.01	0.82	0.77
Alcohol intake (≥20 g/d, %)	32.0	4.9	<0.01	34.4	7.7	<0.01	0.67	0.47
Non-job physical activity (≥2 h/wk, %)	46.0	18.0	<0.01	42.0	23.7	<0.01	0.50	0.36
Dietary intake (/day)								
Vitamin D (µg/1000 kcal)	6.1 ± 3.3	6.7 ± 3.4	0.33	6.2 ± 3.3	6.5 ± 3.6	0.32	0.92	0.80
Fish/shellfish (g)	84 ± 67	84 ± 82	1.00	104 ± 183	73 ± 79	0.03	0.18	0.33
Egg (g)	42 ± 26	31 ± 20	<0.01	33 ± 22	28 ± 18	0.02	<0.01	0.30
Serum 25(OH)D (ng/ml)	28.0 ± 5.1	26.3 ± 4.4	0.03	22.9 ± 7.2	19.4 ± 6.0	<0.01	<0.01	<0.01
Vitamin D deficiency (25(OH)D <20 ng/ml, %)	9.0	9.8	0.86	35.4	62.2	<0.01	<0.01	<0.01
Vitamin D insufficiency (25(OH)D <30 ng/ml, %)	62.0	82.0	<0.01	85.4	94.2	<0.01	<0.01	<0.01

Abbreviation: 25(OH)D, 25-hydroxyvitamin D.

^a^*P* value for the difference between men and women in each workplace. The independent *t*-test was used for continuous variables, and the chi-square test was used for categorical variables.

^b^*P* value for the difference between workplace A and B in each sex. The independent *t*-test was used for continuous variables, and the chi-square test was used for categorical variables.

Table 2 shows multivariate-adjusted mean serum 25-hydroxyvitamin D concentrations in relation to demographic and lifestyle factors according to survey season and sex. In both men and women who donated blood in late autumn, a higher intake of dietary vitamin D was associated with higher serum 25-hydroxyvitamin D concentrations. In the same season, nonsmoking, non-job physical activity, and intakes of fish/shellfish and egg were also associated with higher vitamin D levels among men, whereas serum 25-hydroxyvitamin D concentrations were higher in women aged 35 years or older than in those younger than 35 years. In summer, blood vitamin D levels significantly differed by intake of fish/shellfish and BMI in women. Specifically, serum 25-hydroxyvitamin D concentrations were higher in women with a higher intake of fish/shellfish than in those with a lower intake and lower in obese women than in lean women. In addition, serum 25-hydroxyvitamin D levels tended to increase with vitamin D intake in women, although the relation was not statistically significant. Because few women had a BMI of 23 kg/m^2^ or more, we repeated the analysis using quartiles of BMI for women. However, the results were similar: mean serum 25-hydroxyvitamin D concentrations for the lowest (<18.93 kg/m^2^) and highest (≥22.9 kg/m^2^) quartiles of BMI were 27.3 and 22.5 ng/ml, respectively (*P* = 0.02). In men, alcohol drinkers tended to have higher mean serum 25-hydroxyvitamin D concentrations than did nondrinkers in both seasons.

**Table 2.** Multivariate-adjusted^a^ mean serum 25-hydroxyvitamin D concentration (ng/ml) in relation to demographic and lifestyle factors according to survey season and sex

	Workplace A (July)						Workplace B (November)					
		
	Men (*n* = 100)			Women (*n* = 61)			Men (*n* = 212)			Women (*n* = 156)		
				
	*n*	Mean (SE)	*P* value^b^	*n*	Mean (SE)	*P* value^b^	*n*	Mean (SE)	*P* value^b^	*n*	Mean (SE)	*P* value^b^
Age (years)												
<35	22	28.4 (1.6)		18	23.7 (2.3)		52	22.9 (1.1)		55	16.9 (1.8)	
35–44	38	28.1 (1.3)	0.83	30	25.2 (2.1)	0.26	75	23.8 (0.9)	0.49	52	19.7 (1.7)	0.02
≥45	40	28.9 (1.2)	0.73	13	25.9 (2.5)	0.31	85	22.3 (1.0)	0.62	49	19.7 (1.7)	0.04
	*P* for interaction = 0.45						*P* for interaction = 0.07					
Body mass index (kg/m^2^)												
<21	14	27.2 (1.8)		38	26.9 (2.2)		52	22.9 (1.1)		84	18.3 (1.6)	
21–22.9	27	28.6 (1.4)	0.42	14	28.0 (2.5)	0.43	58	23.0 (1.1)	0.90	31	18.2 (1.8)	0.97
23–24.9	20	29.9 (1.4)	0.14	2	22.6 (3.8)	0.23	41	24.3 (1.2)	0.31	21	19.1 (1.9)	0.59
≥25	39	28.2 (1.3)	0.54	7	22.3 (2.1)	0.04	61	21.9 (1.1)	0.46	20	19.6 (2.1)	0.40
	*P* for interaction = 0.25						*P* for interaction = 0.17					
Occupation												
Office work	93	27.5 (0.7)		34	24.5 (2.2)		185	22.8 (0.6)		111	18.7 (1.6)	
Others	7	29.5 (2.0)	0.32	27	25.3 (2.1)	0.51	27	23.2 (1.3)	0.78	45	18.8 (1.7)	0.96
	*P* for interaction = 0.32						*P* for interaction = 0.89					
Smoking												
Nonsmoker	57	29.2 (1.2)		59	24.4 (1.2)		118	24.6 (0.9)		152	19.3 (0.8)	
Current smoker	43	27.8 (1.3)	0.20	2	25.5 (3.5)	0.74	94	21.4 (0.9)	0.002	4	18.2 (3.0)	0.74
	*P* for interaction = 0.89						*P* for interaction = 0.64					
Alcohol drinking												
Nondrinker	16	26.6 (1.6)		18	25.5 (2.0)		28	22.1 (1.4)		54	18.5 (1.8)	
<20 g/day	52	29.6 (1.2)	0.046	40	25.9 (1.9)	0.77	111	22.0 (0.9)	0.98	90	19.5 (1.7)	0.35
≥20 g/day	32	29.3 (1.4)	0.12	3	23.3 (3.4)	0.48	73	25.0 (1.0)	0.07	12	18.3 (2.1)	0.92
	*P* for interaction = 0.55						*P* for interaction = 0.20					
Non-job physical activity												
None	34	29.0 (1.4)		33	24.0 (2.1)		77	20.9 (0.9)		78	19.2 (1.6)	
<2 hours/week	20	29.5 (1.5)	0.76	17	25.0 (2.3)	0.46	46	23.6 (1.2)	0.04	41	19.0 (1.7)	0.86
≥2 hours/week	46	27.0 (1.3)	0.11	11	25.8 (2.5)	0.39	89	24.5 (1.0)	0.002	37	18.0 (1.9)	0.32
	*P* for interaction = 0.10						*P* for interaction = 0.004					
Vitamin D intake (µg/1000 kcal)												
T1 (<4.54)	36	29.8 (1.3)		16	23.7 (2.3)		73	20.9 (1.0)		51	17.2 (1.7)	
T2 (4.54–<7.0)	34	27.5 (1.4)	0.07	27	24.7 (2.3)	0.49	64	23.5 (1.0)	0.03	52	19.3 (1.7)	0.08
T3 (≥7.0)	30	28.3 (1.4)	0.27	18	26.4 (2.1)	0.11	75	24.6 (1.0)	0.001	53	19.7 (1.8)	0.04
	*P* for interaction = 0.04						*P* for interaction = 0.83					
Fish/shellfish intake^c^ (g/day)												
T1 (<44.4)	28	28.1 (1.5)		20	22.1 (2.2)		68	21.2 (1.0)		60	17.9 (1.6)	
T2 (44.7–<83.0)	34	29.1 (1.3)	0.47	20	24.3 (2.1)	0.11	66	22.8 (1.0)	0.19	56	20.2 (1.8)	0.052
T3 (≥83.0)	38	28.7 (1.4)	0.69	21	26.4 (2.0)	0.006	78	24.7 (1.0)	0.003	40	19.6 (1.8)	0.20
	*P* for interaction = 0.12						*P* for interaction = 0.48					
Egg intake^d^ (g/day)												
T1 (<21.3)	25	28.5 (1.5)		23	25.2 (2.2)		59	21.6 (1.1)		68	17.8 (1.7)	
T2 (21.4–<37.8)	28	27.5 (1.5)	0.50	17	24.8 (2.2)	0.78	86	23.1 (0.9)	0.20	40	19.2 (1.8)	0.24
T3 (≥42.4)	47	29.3 (1.2)	0.55	21	26.5 (2.2)	0.41	67	24.1 (1.0)	0.04	48	19.3 (1.7)	0.19
	*P* for interaction = 0.77						*P* for interaction = 0.71					

^a^Adjusted for other variables, excluding intake of fish/shellfish and egg in this table.

^b^As compared with the first category of the variable.

^c^Adjusted for other variables, excluding intake of vitamin D and egg in this table.

^d^Adjusted for other variables, excluding intake of vitamin D and fish/shellfish in this table.

## DISCUSSION

In this cross-sectional study of apparently healthy Japanese workers, we found a high prevalence of vitamin D deficiency (35% in men and 62% in women) in late autumn, during which dietary vitamin D intake (in both men and women) and nonsmoking and non-job physical activity (in men) were significantly associated with higher serum 25-hydroxyvitamin D concentrations. In summer, high fish/shellfish intake and low BMI were associated with higher serum 25-hydroxyvitamin D concentrations in women. To our knowledge, the present study is the first to examine the season-specific association between lifestyle factors and blood vitamin D levels in an occupational setting.

Studies in Western countries have shown that blood vitamin D levels vary by season and that the prevalence of vitamin D deficiency was higher during winter.^3^^,^^4^^,^^6^^,^^9^^,^^11^^,^^14^^,^^16^ In the present study, the prevalence of vitamin D deficiency and mean serum 25-hydroxyvitamin D concentration were 9.3% and 27.4 ng/ml, respectively, in summer and 46.7% and 21.4 ng/ml in late autumn, which confirms seasonal variation in vitamin D status as well as endemic vitamin D deficiency among a Japanese working population in a season with less sun exposure. Our data conform with the few Japanese studies that have compared blood vitamin D levels in different seasons.^5^^,^^7^^,^^24^ In a study of 197 adult men and women aged 20 to 68 years in the Tokai area (35.3° N) of Japan,^7^ the prevalence of vitamin D deficiency and mean serum vitamin D concentration were 1% and 31.6 ng/ml, respectively, in September and 26% and 23.1 ng/ml in December. Another study reported that mean 25-hydroxyvitamin D_3_ concentration was 31.5 ng/ml in summer and 23.9 ng/ml in winter among 122 adult women aged 45 to 81 years in the Hokuriku area (37.5° N) of Japan.^24^ Another study of 758 adult men and women in the Kansai area (34.5° N) of Japan^5^ reported that the concentration of vitamin D in blood collected in summer was significantly higher than in blood collected in winter. In the present study, serum 25-hydroxyvirtamin D concentrations were measured by using a competitive protein binding assay. There are several types of vitamin D assays, including radioimmunoassay (RIA) and high-performance liquid chromatography (HPLC); however, the best method for reliable and consistent evaluation of blood 25-hydroxyvitamin D levels remains controversial.^33^ Because the vitamin D assay methods used in previous Japanese studies, ie, RIA^7^ and HPLC,^5^^,^^24^ differed from ours, caution should be exercised when comparing the prevalence of vitamin D deficiency and vitamin D concentrations in the present study with those in the literature.

Women had a lower mean serum vitamin D concentration than did men, regardless of the season of blood collection. This sex difference has been observed in many,^3^^–^^9^ but not all, studies.^12^^,^^14^^,^^16^^,^^21^ Although the reason for the discrepancy is unclear, it probably reflects greater avoidance of sunlight among women. Sun avoidance includes the use of sunscreens^34^^,^^35^ that effectively absorb ultraviolet β radiation,^2^ thus resulting in reduced vitamin D production in the skin. Sex differences in vitamin D status might also be due to differences in body composition profiles. Vitamin D is fat-soluble and is thus stored in fat tissue,^36^ the level of which is generally higher in women than in men. This results in lower blood vitamin D in women.

With regard to lifestyle factors, we found significant associations of serum vitamin D concentration with smoking and physical activity (in men) and vitamin D intake (in both men and women) in late autumn. In summer, fish/shellfish intake was significantly associated with serum vitamin D concentration, and serum vitamin D levels tended to increase with vitamin D intake in women only. The differential association by season suggests that lifestyle factors have a significant role in determining vitamin D status in darker seasons, whereas their effects are limited during sunny seasons.

Our findings regarding smoking agree with those of a study that analyzed data by season and showed that smokers had lower circulating vitamin D levels as compared with nonsmokers in winter, spring, and fall, but not summer.^6^ Data suggesting that smoking lowers vitamin D concentrations have also been reported in studies that adjusted for season of blood collection^14^^,^^18^ and in those conducted in summer^13^ and winter.^15^^,^^17^ The mechanism linking smoking to decreased vitamin D status is unclear, but Brot et al^15^ speculated that smoking alters hepatic metabolism of 25-hydroxyvitamin D due to its harmful compounds, which include tar, nicotine, and heavy metals.

Because systemic vitamin D status is determined largely by the amount of vitamin D produced in skin due to sunlight exposure,^2^ people who habitually participate in outdoor activities would be expected to have higher vitamin D levels than those who do not participate in such activities. In fact, physical activity^6^^,^^12^^,^^14^^,^^18^ and outdoor physical activity^9^ have been shown to be associated with higher levels of vitamin D in blood in some but not all studies, after adjustment for seasonal variation.^4^^,^^15^ In the present study, we found an association between non-job physical activity and vitamin D status in men in late autumn only, suggesting a beneficial role for leisure-time sunlight exposure in the maintenance of better vitamin D status when sunlight is relatively weak. Using data from the US Third National Health and Nutrition Examination Survey, Scragg and Camargo^8^ found an association between outdoor physical activity and higher serum 25-hydroxyvitamin D concentrations in all seasons. Due to the survey procedure, however, the data may not be suitable for assessing the modifying effect of season on the association between lifestyle and vitamin D status. Our finding of a null association in summer may be due to insufficient information regarding both the location of physical activity (indoors or outdoors) and sunlight exposure during work. Alternatively, the lack of an association might be because, in summer, daily sunlight exposure induces considerable vitamin D production in the skin.^2^ Thus, additional exposure to sunlight during leisure-time physical activity might not further increase vitamin D levels. We also observed no association between non-job physical activity and serum 25-hydroxyvitamin D among women in November. As mentioned above, this might be due to sunlight avoidance, including the use of sunscreens that result in reduced vitamin D production in skin.

Interestingly, male alcohol drinkers tended to have higher mean vitamin D concentrations than did nondrinkers in both seasons. A similar finding was reported in some,^4^^,^^6^ but not all, previous studies.^3^^,^^15^^,^^18^ In an animal study, ethanol administration significantly increased 25-hydroxyvitamin D_3_ content in plasma and liver, but decreased body reserves of vitamin D_3_ in plasma, muscle, and adipose tissue,^37^ which suggests that circulating 25-hydroxyvitamin D concentration is not a reliable marker of systemic vitamin D status during chronic ethanol administration.^37^ If the same mechanism is present in humans, our findings should be interpreted cautiously.

As regards dietary intake, several studies have reported a positive association of vitamin D concentration with dietary intake of vitamin D^4^^,^^6^^,^^14^^,^^15^^,^^18^^–^^20^ and fish or oily fish.^3^^,^^6^^,^^9^^,^^21^^–^^23^ Given the significant contribution of sun-induced vitamin D in the skin to systemic vitamin D in summer, the association between dietary vitamin D intake might be observable only in seasons with low sun exposure. However, the results of Western studies that analyzed data by season are not entirely consistent with this expectation: blood vitamin D concentrations were positively associated with fish or oily fish intake throughout the year^3^ and in winter only^6^ and with vitamin D intake in all seasons except winter^4^ and predominantly in winter.^19^ In the present study, serum vitamin D concentrations were higher among women with higher intakes of vitamin D and fish/shellfish even in summer, although the finding on vitamin D intake concentration was not statistically significant. This finding suggests that dietary vitamin D intake is still an important determinant of systemic vitamin D status in women, who probably have lower sunlight-induced vitamin D production in the skin, as compared with men, due to sunlight avoidance.

Major strengths of the present study include a high participation rate (91%) and the similar occupational background of the participants. Thus, the possibility of selection bias and confounding by unmeasured variables, including socioeconomic status, is low. Our study also had limitations. First, an association derived from a cross-sectional study does not necessarily indicate causality. However, it is unlikely that 25-hydroxyvitain D status influenced dietary intake and physical activity. Second, we compared the exposure-outcome association between different populations to assess seasonal difference in association. Therefore, the difference in association between 2 seasons might be partly due to the difference between workplaces in known and unknown vitamin D-associated factors that were not measured in our study. Repeated measurements of the same subjects in different seasons might provide additional valid data for that purpose. Third, we had no information on some factors known to influence vitamin D status, including body composition profile, sun avoidance, and skin pigmentation. The inclusion of these factors in the analysis might strengthen or weaken the association. Fourth, because the study participants were employees of municipal offices in Japan, the present findings might not be generalizable to populations with different background characteristics. Finally, because our sample size was not sufficiently large for stratified analysis, the statistical power might be too low to detect a modest association, particularly in women.

In conclusion, we observed a high prevalence of vitamin D deficiency in a Japanese working population in late autumn. Lifestyle factors, including dietary vitamin D intake, smoking (men), and physical activity (men), were significant predictors of serum 25-hydroxyvitamin D concentration in late autumn. In summer, intake of fish/shellfish—a major source of dietary vitamin D—was associated with serum 25-hydroxyvitamin D in women. Research should confirm whether modification of these lifestyle factors improves vitamin D status in seasons with low sun exposure.
